# Population genomics reveal deep divergence and strong geographical structure in gentians in the Hengduan Mountains

**DOI:** 10.3389/fpls.2022.936761

**Published:** 2022-08-25

**Authors:** Peng-Cheng Fu, Shan-Shan Sun, Peter M. Hollingsworth, Shi-Long Chen, Adrien Favre, Alex D. Twyford

**Affiliations:** ^1^School of Life Science, Luoyang Normal University, Luoyang, China; ^2^Royal Botanic Garden Edinburgh, Edinburgh, United Kingdom; ^3^Key Laboratory of Adaptation and Evolution of Plateau Biota, Northwest Institute of Plateau Biology, Chinese Academy of Sciences, Xining, China; ^4^Senckenberg Research Institute and Natural History Museum, Frankfurt, Germany; ^5^Ashworth Laboratories, Institute of Evolutionary Biology, The University of Edinburgh, Edinburgh, United Kingdom

**Keywords:** ancestral range estimation, *Gentiana hexaphylla*, Hengduan Mountains, isolation by distance, plastid, nuclear SNPs

## Abstract

Understanding the evolutionary and ecological processes driving population differentiation and speciation can provide critical insights into the formation of biodiversity. Here, we examine the link between population genetic processes and biogeographic history underlying the generation of diversity in the Hengduan Mountains (HM), a region harboring a rich and dynamic flora. We used restriction site-associated DNA sequencing to generate 1,907 single-nucleotide polymorphisms (SNPs) and four-kb of plastid sequence in species of the *Gentiana hexaphylla* complex (Gentianaceae). We performed genetic clustering with spatial and non-spatial models, phylogenetic reconstructions, and ancestral range estimation, with the aim of addressing the processes influencing diversification of *G. hexaphylla* in the HM. We find the *G. hexaphylla* complex is characterized by geographic genetic structure with clusters corresponding to the South, North and the central HM. Phylogenetic reconstruction and pairwise *F*_ST_ analyses showed deep differentiation between Southern and Northern populations in the HM. The population in Mount Taibai exhibited the highest genetic similarity to the North HM. Ancestral range estimation indicated that the *G. hexaphylla* complex originated in the central HM and then diverged in the Pliocene and the Early Pleistocene, before dispersing widely, resulting in the current distinct lineages. Overall, we found deep genomic differentiation in the *G. hexaphylla* complex corresponds to geographic barriers to dispersal in the HM and highlights a critical role of the uplift of the Daxue Mountains and subsequent climatic fluctuations underlying diversification. The colonization of *G. hexaphylla* in the Mount Taibai region suggests directional dispersal between the alpine flora of the Qinling Mountains and the HM.

## Introduction

Alpine floras, those that grow above the tree line, are particularly species-rich communities enriched with endemic taxa adapted to challenging environments with a short growing seasons and harsh winters. These communities have been profoundly shaped by recurrent cycles of historical climatic change, and continue to be affected by climate as conditions warm. Understanding the evolution of these diverse communities must consider not only past climatic changes, but the full range of processes promoting population divergence, range shifts, and speciation (Antonelli et al., 2018; Muellner-Riehl et al., 2019; Kirschner et al., 2020). In particular, in-depth biogeographic studies must consider cryptic species diversity, which may either be a consequence of *in situ* speciation, colonization by lowland taxa followed by allopatric divergence (local recruitment), or the recurrent immigration of novel and pre-adapted lineages *via* long-distance dispersal (Muellner-Riehl et al., 2019; Ding et al., 2020). Although relevant in all mountain systems of the world, research on these aspects has primarily focused on a few mountainous regions, such as the Hengduan Mountains (HM).

The Mountains of Southwest China, a global hotspot of biodiversity including the HM (Myers et al., 2000; Marchese, 2015), is an ideal area for exploring the spatial–temporal evolution of alpine communities and the drivers underlying speciation and diversification. This region harbors a rich flora with a high proportion of endemics (Wu, 1988; Boufford, 2014) and is characterized by a high rate of *in situ* speciation (Xing and Ree, 2017; Ding et al., 2020). There are estimated to be up to 16,550 species in the HM, accounting for approximately 62% of the total number of seed plant species in China, of which at least 3,300 are endemic (Sun et al., 2017). Moreover, the HM hosts a particularly rich alpine flora with an estimated 3,030 species of alpine seed plants (Sun et al., 2017). Furthermore, the complex topography of the HM, characterized by high ruggedness and deeply dissected landscapes, creates fine-scale environmental heterogeneity that may limit dispersal and subdivide distribution ranges, therefore creating complex population genetic structure and promoting divergence and incipient speciation (Scherrer and Körner, 2011; Li et al., 2021).

Major progress has been made in understanding the general processes shaping the evolutionary history of plants across the HM (Liu et al., 2014; Wen et al., 2014; Favre et al., 2015; Sun et al., 2017; Xing and Ree, 2017; Ding et al., 2020). The diversity of plant species is largely a product of vicariance and postglacial recolonization of alpine plants found in this region (Qiu et al., 2011; Liu et al., 2012; Sun et al., 2017; Muellner-Riehl, 2019), with recent *in situ* diversification in response to local uplift in the HM during the Late Miocene to the Pliocene (Favre et al., 2015; Ding et al., 2020). However, recent comparative analyses of plant species distributions across the region have shown that the HM is not a cohesive entity, but a diverse mosaic of floristic elements shaped by geography, elevation and climate (Li et al., 2021; Muellner-Riehl and Favre, 2021). As such, it is currently unclear how topological changes and changes of connectivity through time within and among each of the seven mountain subranges composing the HM have contributed to species-richness and endemism. The respective role of each of these subranges as regional refugia or sinks is also largely unknown, as case studies often consider the HM as a single entity (Liu et al., 2012; Muellner-Riehl, 2019). Finally, the alpine flora of the HM has not evolved in complete isolation, and adjacent regions such as the Qinling Mountains (QM, 400 km away), which includes Mount Taibai, the highest peak (3,500 m a.s.l.) in Central and East China, may have also been a source for speciation and floristic exchange of species now found in the HM.

The use of high-throughput sequencing is an extremely promising route to elucidate fine-scale genetic structure and the processes underlying speciation among the dynamic flora of the HM (Liu et al., 2014). Of particular value would be to use a large number of nuclear markers, which often provide high-levels of resolution for studying recent species divergence. Recent studies in Europe have emphasized the complex evolutionary history that has shaped the present genetic diversity of refugial populations, and stressed the need to revisit their phylogeographic history with genomic approaches (Dufresnes et al., 2020; Marková et al., 2020). However, genomic data needs to be matched with suitable analytical tools, to account for confounding factors that may obscure evolutionary inference. For example, one challenge is detecting clearly defined genetic units that have evolved independently in different geographic regions, against the background of clinal population structure arising as a result of isolation by distance (IBD) that is a common confounding factor that can obscure the genetic signature of biogeographic barriers (Meirmans, 2012; Perez et al., 2018; Twyford et al., 2020). Such issues can increasingly be accounted for and modelled using appropriate genomic datasets and analytical tools.

*Gentiana* L. (Gentianaceae), a worldwide alpine genus of about 360 species (Ho and Liu, 2001), is a group where general phylogenetic relationships (Favre et al., 2020) and biogeographic history (Favre et al., 2016) are relatively well understood. It was previously shown that the Qinghai-Tibetan Plateau region, including the HM, is the centre of biodiversity for the genus and the primary source area for colonization to other regions (Favre et al., 2016). The HM is home to 135 *Gentiana* species of which 66 are endemics (Yu et al., 2020). Phylogenetic and population genetic analyses indicate that climatic changes and mountain uplift are correlated with recent divergence, speciation and diversification in most clades of *Gentiana* (Zhang et al., 2007, 2009b; Lu et al., 2015; Fu et al., 2018, 2020a, 2021b). However, these previous studies treated the HM as a single geographic entity, and thus our knowledge of the phylogeographic history of the genus in the HM is not well understood at a finer scale.

In this study, we performed population genomic analysis of the gentian species *G. hexaphylla* Maximowicz ex Kusnezow, which belongs to *G.* series *Verticillatae* Marquand, with the aim of investigating fine-scale phylogeographic structure across the topologically complex HM. Previous phylogenetic studies including *G.* series *Verticillatae* indicate very young speciation events and recent radiations (Favre et al., 2016; Fu et al., 2021a), making this an ideal study group for investigating the recent biogeographic history of the region. Using genomic data, we address: (1) whether geographical features (e.g., one or more mountain ranges) in the HM acted as barriers to gene flow, and lead to discrete genetic structure and cryptic lineages; (2) whether changes in distribution range during climate oscillations lead to genetic differentiation and possibly speciation; (3) whether migration occurred between the HM and the nearby but disjunct mountain ranges such as the Qinling Mts.

## Materials and methods

### Study species and sampling

*Gentiana hexaphylla* is a widespread species with phenotypic variation in some characters [e.g., growth habit, leaf number per whorl and corolla colour (Ho and Liu, 2001)], but has only minor morphological differences from some closely related species in series *Verticillatae* (8 species in total, Ho and Liu, 2001), including *G. ternifolia* Franchet, *G. tetraphylla* Maximowicz ex Kusnezow and *G. viatrix* Harry Smith. Thus, we considered these closely related species, often co-occurring with *G. hexaphylla*, to collectively be part of the *G. hexaphylla* complex in this study. The *G. hexaphylla* complex occurs widely across the HM (Ho and Pringle, 1995; Ho and Liu, 2001), tends to reproduce sexually *via* outcrossing in open sunny habitats (Xue et al., 2018), and some species have horticultural value. Despite this interest, taxonomic confusion remains problematic for this group of species. For example, individuals from Mount Taibai have been recognized as *G. hexaphylla* in the *Flora of China* (Ho and Pringle, 1995) but treated as *G. arethusae* by Ho and Liu (2001). These species are very similar, and supposedly differ only by the linear and acuminate upper stem leaves and calyx lobes. Based on numerous field observations, these purported diagnostic traits appear to be highly variable (personal observations) and thus the population from Mount Taibai is treated as *G. hexaphylla* in this study.

**Figure 1:**
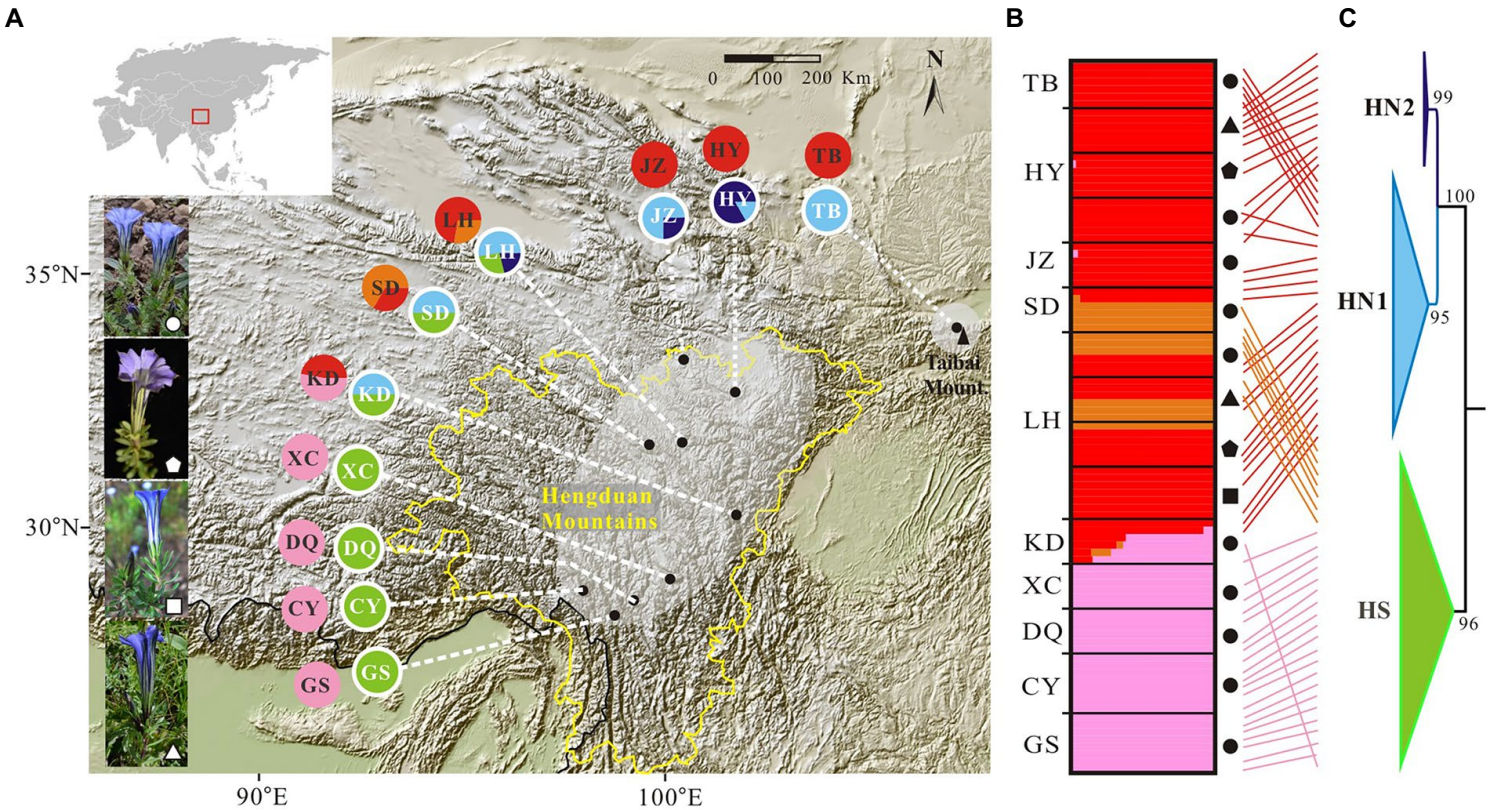
Genetic structure in the *Gentiana hexaphylla* complex. **(A)** Pie charts showing frequency of plastid haplotypes (inner circles) and colour-coded groups based on 1,907 SNPs in FastStructure (outer circles) for each sampling site. Black dots represent sampling sites. Map came from the Institute for Planets. **(B)** Bar plots showing probabilities of ancestral clusters of each sample at *K* = 3 in FastSTRUCTURE. The sampling site is shown on the left of the bar plot. Dark shapes in panels **B**,**C** indicate different species: circles, *G. hexaphylla*; pentagons, *G. viatrix*; squares, *G. tetraphylla*; triangles, and *G. ternifolia*. **(C)** Phylogeny of plastid data based on eight plastome fragments. Support values from the maximum likelihood analyses are shown on the nodes. Subclades are named HN1, HN2, and HS. Line connections indicate the same individual in the phylogeny and bar plot. The border of the Hengduan Mountains is indicated with a broken yellow line, and based on Ding et al. (2020). An equal-area projection has been used in the map. White shapes in the photos indicate different species, as used in panels **B**,**C**. Photographs: Peng-Cheng Fu.

We sampled 15 populations of the *G. hexaphylla* complex from 10 localities throughout its range, collecting a total of 95 individuals (Table 1; Figure 1). Our sampling aimed to maximize the number of sampling sites across the mountain ranges in the HM, in order to test for the presence of geographic barriers. Within sites, we collected multiple individuals to test for taxonomic clustering when two or more species co-occurred. For sequencing, we selected typical individuals characterized by leaf number per whorl ranging from three to six (Fu et al., 2020b). A total of 10 populations of *G. hexaphylla*, two populations of *G. ternifolia*, one population of *G. tetraphylla* and two populations of *G. viatrix* were sampled. Young leaves of each individual were dried in silica gel. Voucher specimens were deposited in the herbarium of Luoyang Normal University.

**Table 1:** Information of samples and genetic diversity in this study.

Region	ID	No.	Voucher ref.	Species	Average leaves in whorl	Location	Longitude/Latitude	Altitude (m/a.s.l)	*H*_o_	*H*_E_	Pi
North	HY	6	Fu2017202	*G. hexaphylla*	6	Hongyuan, SC	102°14′E/32°39’N	3,731	0.0329	0.1861	0.2061
	HYter	6	Fu2017199	*G. ternifolia*	3	Hongyuan, SC	102°14′E/32°39’N	3,731	0.0552	0.1991	0.2201
	HYvia	6	Fu2017201	*G. viatrix*	5	Hongyuan, SC	102°14′E/32°39’N	3,731	0.0406	0.1912	0.2114
	JZ	6	Fu2017229	*G. hexaphylla*	6	Jiuzhi, QH	101°19′E/33°22’N	4,048	0.0382	0.1972	0.2181
	TB	6	Fu2019001	*G. hexaphylla*	6	Taibai, SX	107.967E/33.967 N	3,520	0.0440	0.1994	0.2206
Central	SD	6	Fu2016046	*G. hexaphylla*	6	Seda, SC	100°06′E/31°49’N	4,483	0.0352	0.1828	0.2022
	KD	6	Fu2016087	*G. hexaphylla*	6	Kangding, SC	101°47′E/30°04’N	4,224	0.0478	0.1834	0.2028
	LH	6	Fu2017173	*G. hexaphylla*	6	Luhuo, SC	100°43′E/31°44’N	4,022	0.0550	0.1845	0.2040
	LHter	6	Fu2017170	*G. ternifolia*	3	Luhuo, SC	100°43′E/31°44’N	4,022	0.0418	0.2031	0.2246
	LHtet	7	Fu2017171	*G. teterphylla*	4	Luhuo, SC	100°43′E/31°44’N	4,022	0.0308	0.1572	0.1709
	LHvia	6	Fu2017172	*G. viatrix*	5	Luhuo, SC	100°43′E/31°44’N	4,022	0.0371	0.1873	0.2076
South	DQ	6	Fu2018052	*G. hexaphylla*	6	Deqin, YN	99°04′E/28°20’N	4,326	0.0514	0.1940	0.2146
	GS	8	Fu2018064	*G. hexaphylla*	6	Gongshan, YN	98°45′E/28°04’N	3,900	0.0495	0.1756	0.1903
	CY	8	Fu2018088	*G. hexaphylla*	6	Chayu, TB	98°04′E/28°36’N	4,330	0.0485	0.1695	0.1835
	XC	6	Fu2016156	*G. hexaphylla*	6	Xiangcheng, SC	100°03′E/28°49’N	4,628	0.0485	0.1649	0.1823

*H_O_*, average observed heterozygosity; *H_E_*, average heterozygosity; Pi, nucleotide diversity. Abbreviation after localities indicate provinces as follows: QH, Qinghai; SC, Sichuan; SX, Shaanxi; TB, Tibet; YN, Yunnan.

### Library construction, sequencing, and SNP calling

Our genomic sequencing approach aimed to generate many unlinked nuclear SNPs to infer fine-scale population clustering and to estimate genetic divergence, as well as to recover plastid genomic DNA sequence to perform phylogenetic and molecular dating analyses.

Total genomic DNA was extracted from dry leaves using a Dzup plant genomic DNA extraction kit (Sangon, Shanghai, China). DNA concentrations were quantified with a Qubit 2.0 fluorometer (Life Technologies). For RAD library construction and sequencing (Miller et al., 2007), each sample was digested with the restriction enzyme *EcoR*I followed by the ligation of the P1 adapter by T4 ligase. Fragments were pooled, randomly sheared and size-selected to 350–550 bp. A second adapter (P2) was then ligated. The ligation products were purified and PCR-amplified, followed by gel purification and size selection for fragments in the range of 350–550 bp. Libraries were multiplexed and sequenced using 2 × 150 bp reads generated on the Illumina Novaseq 6,000 (Tianjin, China).

Samples were initially de-multiplexed with the *process_radtags* script in Stacks 2.0 (Catchen et al., 2011, 2013). Raw reads were filtered and trimmed with Trimmomatic 0.32 (Bolger et al., 2014) with default parameters, to remove adaptor sequences and low-quality reads and sites, and then checked for quality with FastQC 0.11.2. We used Stacks 2.0 (Catchen et al., 2011, 2013) to identify orthologous loci across individuals. Clean sequences were assembled *de novo* using *denovo_map*, with a minimum stack depth of three (*m* = 3), and we tested a range of different mismatches between stacks within and between individuals (M = *n* = 2, 3 or 4). PCR duplicates were filtered using *gstacks* following the approach of Rochette et al. (2019). At least 75% of individuals in a population were required to retain a locus (−r 0.75), and SNPs identified in all individuals with a minor allele frequency (MAF) of less than 5% were removed (−min-maf 0.05). SNPs with a missing frequency of less than 50 % among individuals (−max-missing 0.5) were retained using vcftools 0.1.13 (Danecek et al., 2011). Linkage-disequilibrium (LD) SNP pruning was performed in vcftools to excludes variants from closer than 100 bp (−thin 100). Heterogeneous loci were filtered out in TASSEL 5 (Bradbury et al., 2007) to exclude SNPs originating from putative paralogs. We estimated genetic diversity indices including nucleotide diversity (Pi), expected heterozygosity (*H_E_*) and observed heterozygosity (*H_O_*) using the *populations* module in Stacks.

To obtain plastid sequences, clean reads were assembled using the GetOrganelle pipeline (Jin et al., 2020) with default parameter. We used the published plastome of *G. hexaphylla* (MG192305; Sun et al., 2018) as the reference. Contigs longer than 500 bp were mapped back to the plastome of *G. hexaphylla* in Geneious Basic 5.6.4 (Kearse et al., 2012). Shared plastome regions present in at least one individual per population were extracted, aligned using MAFFT (Katoh et al., 2002) and then concatenated for downstream analyses.

### Population genetic structure

To assess the levels of genetic differentiation between populations, we estimated pairwise *F*_ST_ based on nuclear genomic SNPs using the Weir and Cockerham method (Weir and Cockerham, 1984) in vcftools 0.1.13 (Danecek et al., 2011). Pairwise *F*_ST_ values were graphically displayed with the package “pheatmap” using R for Statistical Computing (v. 4.0.1; R Core Team, 2020). Analysis of Molecular Variance (AMOVA) was conducted with GenAlEx 6.5 (Peakall and Smouse, 2006). We tested for IBD (Wright, 1943) by applying a Mantel test using the geographic distance and pairwise genetic distance with zt (Bonnet and Van de Peer, 2002).

For exploring the genetic clusters present within the *G. hexaphylla* complex, we used a Bayesian clustering method implemented in FastSTRUCTURE (Raj et al., 2014) based on the nuclear SNPs identified above. Following Raj et al. (2014), we used the *chooseK.py* script to assess model complexity for the data. Graphical representation of individual cluster assignments was performed using DISTRUCT 1.1 (Rosenberg, 2004). As FastSTRUCTURE makes a number of assumptions, such as individuals being in HWE, we also used a non-model-based method in DAPC (Jombart et al., 2010) in the R package “adegenet” to identify genetic clustering. The most likely K value was selected using Bayesian information criterion (BIC; Jombart et al., 2010). Given the strong signal of IBD in our study (see “Results”), we used an additional spatial method implemented in the R package conStruct to infer patterns of genetic structure considering the geographic distance among the sampled populations (Bradburd et al., 2018). This method can be run without spatial information, which will give results similar to other Bayesian admixture approaches, or with spatial information, where it then explicitly accounts for allele frequency differences as expected by IBD, and can therefore reveal discontinuous genetic variation corresponding to barriers to dispersal. We ran a cross-validation analysis with five replicates, comparing the spatial and non-spatial models with *K* = 1 through 10 for each replicate. Layer contributions were also calculated to interpret cross-validation results. Each training partition (one per replicate) was created by randomly subsampling 90% of the total number of loci and run for 1,000 MCMC iterations.

### Phylogenetic inference and divergence time estimation

We constructed a phylogenetic tree based on the nuclear genomic SNPs using maximum likelihood (ML) in IQ-TREE (Nguyen et al., 2015) with 1,000 replicates. To investigate population relationships and model historical migration events, we used TreeMix 1.2 (Pickrell and Pritchard, 2012) using the SNP data. We calculated the percentage of variation explained by the TreeMix analyses with between 0 and 10 migration events using the treemixVarianceExplained scripts in the RADpipe package.^1^

Using concatenated plastid sequences, ML phylogenetic analyses were conducted with IQ-TREE (Nguyen et al., 2015) implemented in PhyloSuite platform (Zhang et al., 2020) with 1,000 replicates. The substitution model was detected in ModelFinder (Kalyaanamoorthy et al., 2017). Information about outgroup samples is presented in Supplementary Table S1.

We estimated the divergence times of plastid sequences using the Bayesian method implemented in BEAST 2.4 (Drummond et al., 2012; Bouckaert et al., 2014) under the HKY substitution model (as the best model detected in ModelFinder, Kalyaanamoorthy et al., 2017), the Yule model, and the strict clock model. We constrained one of the nodes with the fossil of *G.* section *Cruciata* (*G. cruciata* L. in Germany, Mai & Walther, 1988; the early Miocene, Mai, 2000) following Fu et al. (2021a), namely with lognormal priors with an offset at 16.0 Ma, a mean of 1.0, and a standard deviation of 1.0. Because the fossil record is very limited for gentians, we also employed a secondary calibration approach, constraining the *Gentiana* crown age with the estimated divergence time from Janssens et al. (2020), using uniform priors with a lower age of 21.25 Ma and an upper age of 38.21 Ma (Fu et al., 2021a). We ran three independent MCMC chains with 10,000,000 generations, sampling every 1,000th generation and discarding the initial 10% as burn-in. Convergence was confirmed in TRACER 1.5^2^ and judged by ESS values (>200). Trees were summarized using TreeAnnotator 1.7.5 (Drummond et al., 2012) and visualized in FigTree 1.4.^3^

### Ancestral range estimation and species distribution modelling

We used the R package ‘BioGeoBEARS’ (Matzke, 2013, 2014) to compare biogeographical models and estimate the evolution of geographic ranges across the phylogeny which we obtained using BEAST. The distribution of the four lineages of *G. hexaphylla* were coded for their presence/absence in the four biogeographical regions, which were based on the genetic clusters identified above. Dispersal was restricted to adjacent areas and the maximum range size was set to four, which means no extant cluster can occur in more than the four biogeographical regions. We did not specify an outgroup as the aim of our preliminary analyses was to determine dispersal routes below the species level. We compared six models: dispersal-extinction-cladogenesis (DEC; Ree and Smith, 2008), dispersal-vicariance analysis (DIVA; Ronquist, 1997) and BAYAREA models (Landis et al., 2013), plus all three models separately with the possibility of founder events (+j; Matzke, 2013, 2014). As our main objective was to trace ancestral areas rather than to infer the diversification dynamics or the speciation mode, concerns raised by Ree and Sanmartín (2018) on the DEC + J model are unlikely to have significant effects on our results. The best model was selected using the Akaike information criterion (AIC) and the sample size corrected AIC (AICc) after computing the loglikelihood score (Dupin et al., 2016). The probabilities of the ancestral states at all nodes in the phylogeny were estimated using the best model.

Current distribution data for *G. hexaphylla* included observations made during the course of our fieldwork, and those available in the Global Biodiversity Information Facility (GBIF).^4^ Records occurring less than 10 km from each other were removed in ArcGIS 10.2 in order to avoid multicollinearity. The 19 bioclimatic variables at present, mid-Holocene (6 kya) and LGM (Last Glacial Maximum, ~22 kya) were obtained from the WorldClim dataset (Hijmans et al., 2005) with a spatial resolution of 2.5 arc-min. To avoid multicollinearity, a Pearson correlation analysis of the 19 variables was conducted using SPSS 20. Highly correlated variables with correlation coefficients significantly larger than 0.8 (*p* < 0.05) were removed. MaxEnt 3.4.1 (Phillips et al., 2005) was then applied to predict the potential distribution area. We used 75% of the location data for training and the remaining 25% to test the predictive ability of the model. Effectiveness of the model was evaluated using the receiver operating characteristic (ROC) and the area under the ROC curve (AUC) > 0.9.

## Results

### Data preprocessing and SNP calling

Illumina sequencing of RAD libraries produced an average of 1.83 × 10^7^ reads per sample. After quality filtering the number of reads retained per sample varied from 4.45 × 10^6^ to 8.07 × 10^7^, with a median value of 1.81 × 10^7^ (Supplementary Table S2). A total of 202,861, 229,804, and 257,315 SNPs were called with the three different Stacks parameter settings of M = n set to 2, 3, and 4, respectively, suggesting broadly similar number of SNPs are recovered regardless of the number of mismatches allowed within (M) or between (n) individuals. After filtering for MAF, LD, missing data and heterogeneous loci, the total number of unlinked SNPs obtained with Stacks for all samples was 1,988, 1,875, and 1,907 when M = n was 2, 3, and 4, respectively.

### Population genetic structure and genetic divergence

**Figure 2:**
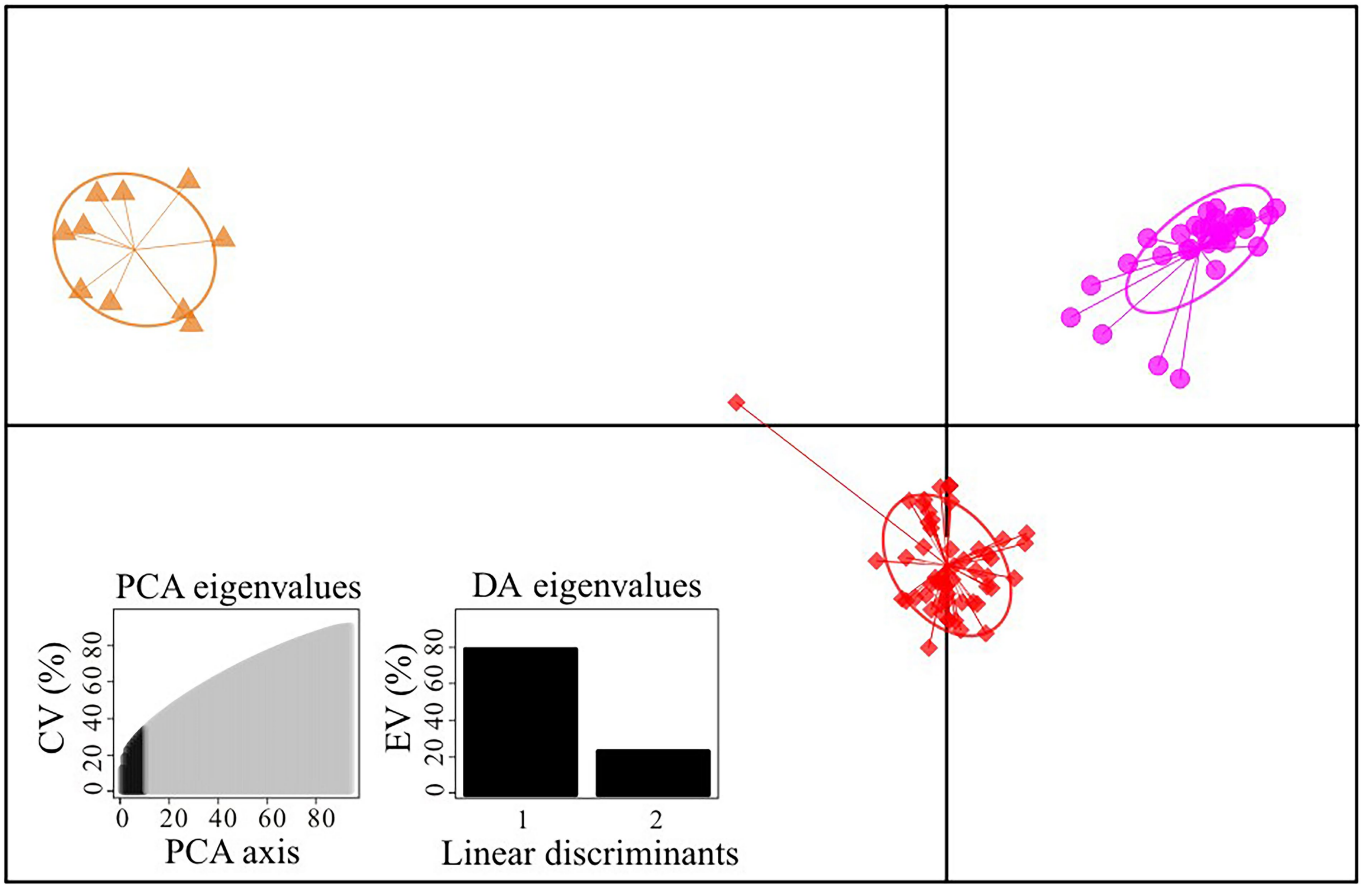
Scatterplot of DAPC analysis in the *Gentiana hexaphylla* complex using 1,907 SNPs. Clusters and inertia ellipses are shown in different colours, consistent with Figure 1. Each dot represents an individual. Insets show histograms of discriminant analysis eigenvalues. CV, cumulated variance; EV, explained variance.

We firstly used the data set of 1,907 SNPs (*m* = 3, M = *n* = 4) to infer population genetic structure in the *G. hexaphylla* complex. *chooseK.py* indicated that the marginal likelihood scores from FastSTRUCTURE analyses peaked at *K* = 3 (Supplementary Figure S1). Generally, the inferred genetic groups were consistent with known geographic barriers present between populations (Figure 1). The northern genetic cluster (TB, JZ, and HY) occurs from the Qionglai Mountains to Mount Taibai and the southern genetic cluster (XC, DQ, GS and CY) from the Shaluli Mountains to Gaoligong Mountains (coloured red and pink, respectively; Figures 1A,B). Populations LH, SD, and KD in the Daxue Mountains in the central HM, was composed of two genetic clusters, and populations LH and SD also formed another genetic cluster (indicated in orange in Figure 1). DAPC analyses suggested an optimal clustering value of *K* = 3 (Supplementary Figure S2), with population groupings corresponding to their geographic location (Figure 2). None of the four study species consistently formed a separate cluster in both FastSTRUCTURE and DAPC analyses. The other two sets of SNPs (1,988 and 1,875 SNPs) gave almost identical results to the dataset of 1,907 SNPs (Supplementary Figure S1, S2), suggesting these inferences are robust to the parameters in Stacks used to assemble reads into loci, thus we performed all downstream analyses on this dataset.

**Figure 3:**
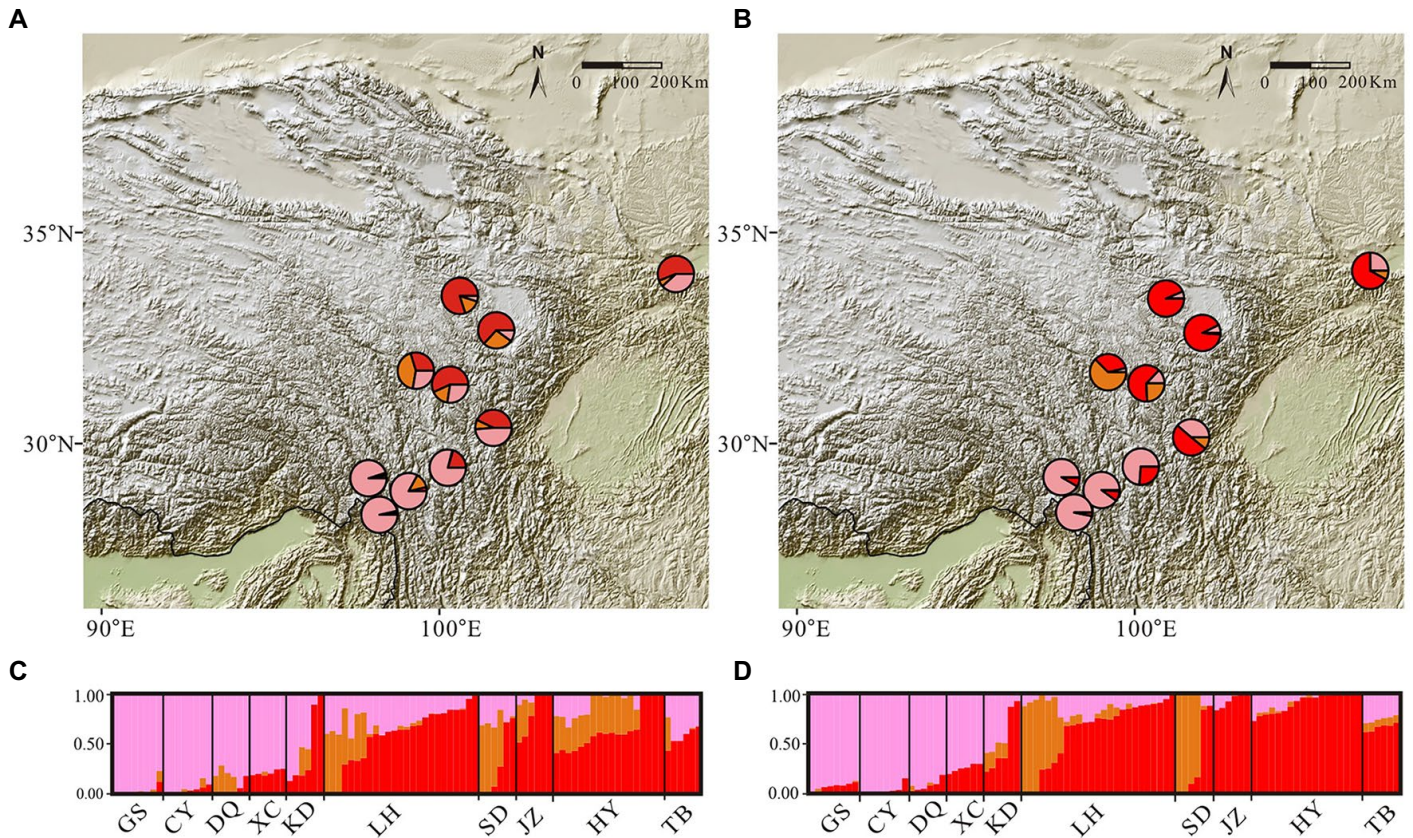
Maps of admixture proportions and bar plots for the *Gentiana hexaphylla* complex inferred using conStruct spatial and non-spatial analyses, using a K-value of three. Pies show mean admixture proportions across individuals from sampling sites inferred using spatial (panel **A**) and non-spatial (panel **B**) models. Admixture bar plots show genetic clustering in spatial (panel **C**) and non-spatial (panel **D**) models. Each bar represents an individual. The sampling sites are labelled at the bottom of the bar plots.

Mantel tests between genetic and geographical distance showed a significant correlation (*R* = 0.49, *p* = 0.0012), indicating strong IBD. The cross-validation analyses in conStruct indicated that the spatial models always had higher predictive accuracy than non-spatial models, with little increase in accuracy when K was greater than three (Supplementary Figure S3). Comparing parametric covariance contributions of each model, layers larger than three generally contributed little to overall covariance across the replicates (Supplementary Figure S3), and are therefore unlikely to be of biological importance. We thus chose three layers for further characterization. In this model, spatial analyses showed genetic clusters separating the northern and southern populations (Figure 3), supporting divergence caused by barriers to dispersal rather than simply IBD in the *G. hexaphylla* complex.

**Figure 4:**
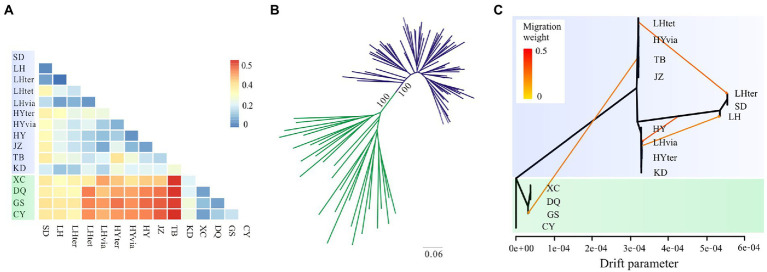
Genetic differentiation and phylogenetic relationships based on genomic SNPs in the *Gentiana hexaphylla* complex. **(A)** Heatmap of Weir and Cockerham’s *F*_ST_ between each pair of populations. **(B)** Unrooted maximum likelihood tree. **(C)** TreeMix graph showing population splits, and migration edges with migration weights indicated by the colour. The x-axis is the drift parameter reflecting the amount of genetic drift that has occurred between populations and the assumed common ancestral population. Locations in the South HM are showed in green and locations in other regions in blue.

The southern populations (XC, DQ, GS, and CY) had higher values of pairwise *F*_ST_ (0.259–0.541) than the northern populations (−0.056–0.320; Figure 4A; Supplementary Table S3). Population (TB) in Mount Taibai had lower values of pairwise *F*_ST_ with the northern than with the southern populations, and the population KD in the Central HM had low values of pairwise *F*_ST_ with both the southern and the northern populations. Populations from the South HM generally had lower nucleotide diversity than other regions (Table 1). Genetic variance mainly occurred among populations (56%) and within populations (37%) rather than among the South, Central and North regions (7%).

### Phylogenetic inference and estimation of divergence time

Using genomic SNPs, the ML tree showed that individuals from the North and the South were assigned to two distinct clades with full support (Figure 4B). None of the species in the *G. hexaphylla* complex formed a monophyletic lineage. The TreeMix analysis also showed that the southern populations were a distinct clade with populations placed on a long branch (Figure 4C). When no migration was allowed, the variance explained was high (99.17%), indicating a simple model of divergence without migration is a good fit to populations of the *G. hexaphylla* complex. Adding migration events in the phylogeny produced a marginally better fit (Supplementary Figure S4), and showed some potential historical dispersal events across the northern and central populations as well as to the southern populations (Figure 4C).

Mapping contigs from each sample to the plastome of *G. hexaphylla* generated eight regions shared among 62 individuals, which covered all populations, and these were concatenated for downstream analyses. The eight aligned regions ranged from 374 bp to 798 bp in length and the aligned concatenated sequences were 4,341 bp. The ML tree indicated that the *G. hexaphylla* complex had two highly supported clades corresponding to the South (HS) and the North (HN), respectively (Figure 1C; Supplementary Figure S5). The HN clade was further divided into two subclades (HN1 and HN2) with high support. Comparing genetic clustering based on genomic SNPs and plastid data showed that samples in the same group from the south and the north were included in the HS and the HN clade, respectively, while the samples in a distinct clade from the central HM were included in the HN clade (Figures 1B,C; Supplementary Table S4). Bayesian inference in BEAST supported the two subclades in the North HM (HN1 and HN2) and two subclades in the South HM (HS and KD; Supplementary Figure S6). More than one lineage co-occurs at the Central and the North HM (Figure 1A).

Our divergence time analyses based on plastid sequences (Supplementary Figure S6) showed that the HN and HS clades in *G. hexaphylla* diverged in the Pliocene, approximately 4.01 Ma (95% HPD: 3.04–5.06 Ma). The two subclades in the HN and HS diverged in the Early Pleistocene 1.71 Ma (95% HPD: 1.09–2.38 Ma) and in the Late Pliocene 3.10 Ma (95% HPD: 2.28–3.88 Ma), respectively.

### Ancestral range estimation and palaeo-distributional reconstruction

**Figure 5:**
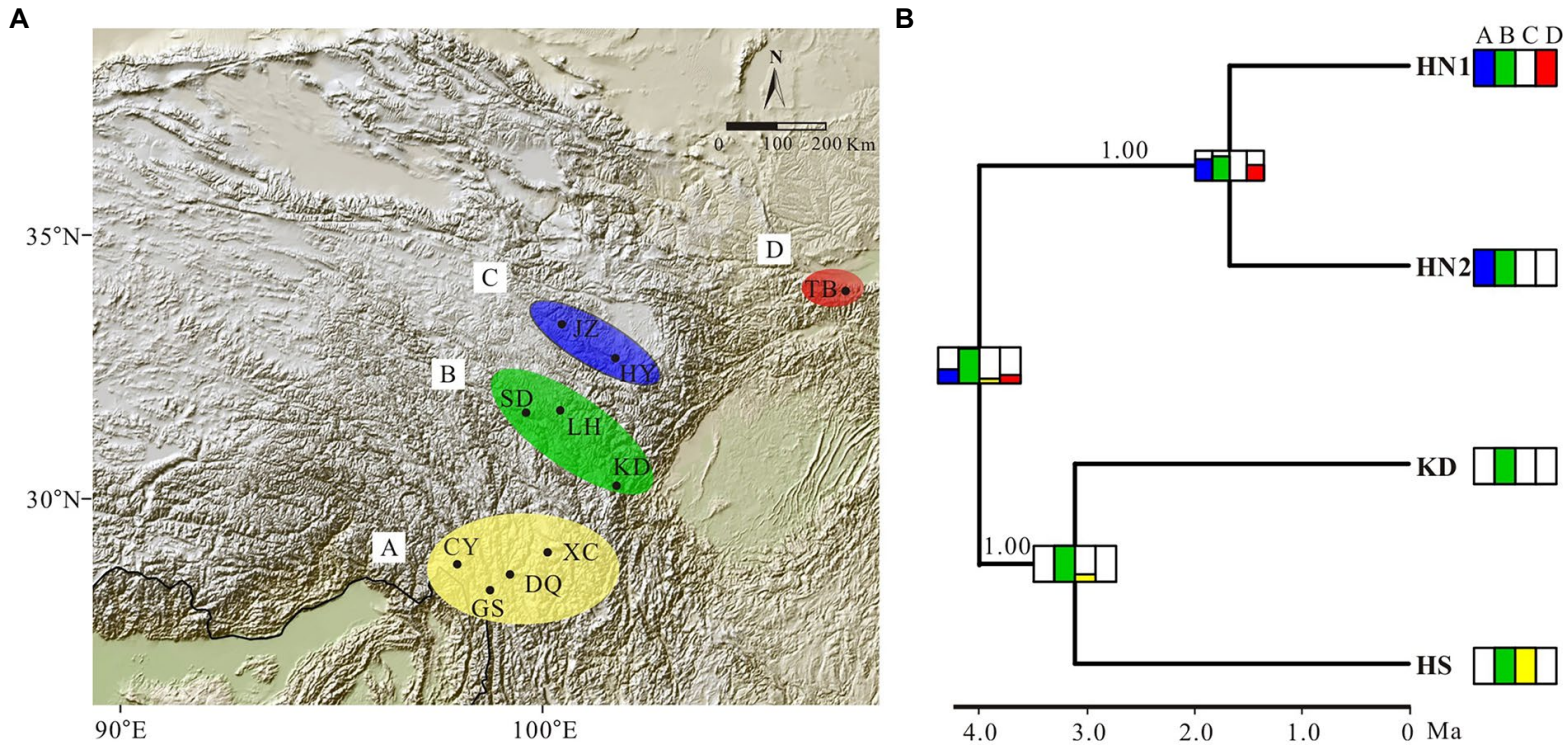
Ancestral area estimation of the *Gentiana hexaphylla* complex based on BioGeoBEARS. **(A)** Area definition for ancestral area estimation, based on the extant distribution of the *G. hexaphylla* complex. **(B)** Ancestral range estimation based on BAYAREALIKE model implemented in BioGeoBEARS based on the result from BEAST. Extant species distributions used for the ancestral area estimations are provided in coloured boxes next to each lineage. Boxes on each node represent the cumulative probabilities for the estimated ancestral range. Phylogenetic support values for Bayesian inference are shown on branches.

Estimation of the evolution of geographic range in BioGeoBEARS indicated that the BAYAREA model was the best fit to the *G. hexaphylla* complex, as it received the largest LnL value and the lowest AIC and AICc scores (Table 2). The DEC model also gave a very similar evolutionary scenario to the BAYAREA model. Based on the probability of each estimated ancestral range, the earliest common ancestor of the four lineages in *G. hexaphylla* complex might have occurred in the central HM (the green area, b) around 4 Ma, implying that the ancestral lineage dispersed out of this area southward and northward independently, which gave rise to different sub-lineages (Figure 5).

**Table 2:** Model comparison and parameters (d, dispersal; e, extinction; j, founder speciation) of ancestral area estimation of the *Gentiana hexaphylla* complex based on BioGeoBEARS.

Model	LnL	numparams	d	e	j	AIC	AIC_wt	AICc	AICc_wt
DEC	−8.39	2	0.17	1.00E^−12^	0	20.77	0.23	32.77	0.31
DEC + J	−8.39	3	0.17	1.00E^−12^	1.00E^−5^	22.77	0.08	Inf	0
DIVALIKE	−9.09	2	0.23	1.00E^−12^	0	22.19	0.11	34.19	0.15
DIVALIKE+J	−9.09	3	0.23	1.00E^−12^	1.00E^−5^	24.19	0.04	Inf	0
**BAYAREALIKE**	**−7.84**	**2**	**0.12**	**0.19**	**0**	**19.68**	**0.39**	**31.68**	**0.54**
BAYAREALIKE+J	−7.84	3	0.12	0.19	1.00E^−5^	21.68	0.14	Inf	0

The optimal model for the *G. hexaphylla* complex is shown in bold.

**Figure 6:**
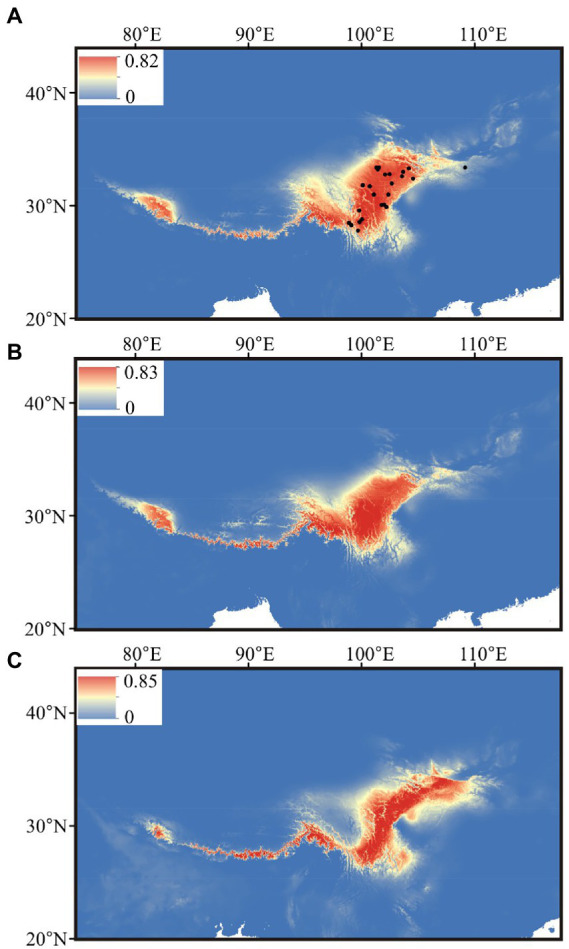
Estimated climatic niches for *Gentiana hexaphylla* in the Qinghai-Tibetan Plateau. Maps are shown at **(A)** present, **(B)** mid-Holocene (~6 kya), and **(C)** LGM (~22 kya). The value of predicted habitat suitability is indicated by the bars in each panel. Current distribution records are shown in the present map by black dots.

After the Pearson correlation analysis, nine bioclimatic variables (bio1–bio4, bio7, bio12–bio15) were kept for distributional reconstruction. The palaeo-distributional reconstruction showed that during the LGM the potential habitat of *G. hexaphylla* was restricted to the Himalayas and the South to East of the HM (Figure 6). Afterwards, from the LGM to today, its range significantly expanded into the HM, but experienced a slight contraction in the Northeast.

## Discussion

As the longest continuously existing alpine flora (Ding et al., 2020), the HM alpine flora offers an excellent opportunity to explore spatial–temporal changes in the distribution ranges of species. Such studies can test how range changes may have been driven by geological or climatic modifications and resulted in divergence, speciation and ultimately diversification. Using genomic data for a common *Gentian* species, we detected deep genetic divergence corresponding to geological barriers in the HM, with divergence likely promoted by both mountain uplift and climatic fluctuations. While we detected divergence in populations spread across the landscape, there was notably high genetic similarity between populations in Mount Taibai and the North HM, indicating a connection between the alpine flora of the Qinling Mts and the HM. Here, we discuss our results in terms of the biogeographic history of this important hotspot for alpine species, and consider the implications for future studies.

### Strong geographic genetic differentiation between the North and South HM

The HM is well known for its extraordinary diversity and high rate of *in situ* alpine speciation (Sun et al., 2017; Xing and Ree, 2017; Ding et al., 2020). However, how genetic subdivision of populations corresponds to major geological features within the HM and surrounding areas remains to be characterized in detail. To address this issue, studies of fine-scale population divergence across this region are necessary, but they have been hampered by the poor resolution offered by traditional markers and/or by the complex evolutionary history of this region (Qiu et al., 2011; Liu et al., 2012; Muellner-Riehl, 2019). Using genomic data, our results clearly show that deep genetic differentiation in the *G. hexaphylla* complex occurs between the North and South HM, two areas which are geographically separated by the Daxue Mountains. Although we detected a significant pattern of IBD, once geographic distance is accounted for, we confirmed that there are clearly defined geographic genetic clusters rather than structure corresponding to a cline (Twyford et al., 2020). Our genomic data showed much clearer genetic structure and geographical divergence in the *G. hexaphylla* complex than previous work on the same species complex using one plastid fragment but with denser population sampling (Fu et al., 2020b). Recently, other genomic studies in plants of the HM, including *Pinus armandii* (Liu et al., 2019) and the *Rheum palmatum* complex (Feng et al., 2020), have also found similar results. Together with other studies (e.g., Dufresnes et al., 2020; Marková et al., 2020), these results show how genomic data can resolve complex and potentially cryptic genetic patterns, even in topographically complex settings.

The deeply dissected landscape of the HM is expected to generate numerous barriers to gene flow and thus to promote genetic differentiation and speciation. This was supported by the high *F*_ST_ value in our study, as well as the high variance explained by the TreeMix model without migration, as well as by the presence of private haplotypes in previous work (Fu et al., 2020b). Our finding of a North–South divide in the HM is in line with others studies, for example in *Pinus armandii* (Liu et al., 2019) and the *Rheum palmatum* complex (Feng et al., 2020). In addition, changes in species richness and composition can be observed across this zone (Zhang et al., 2009a). Thus, the Daxue Mountains, which create a North–South divide, is the primary geological barrier for this species complex. To the contrary, our study did not detect the Nu River—the notorious Salween-Mekong divide isolating the Gaoligong Mountains (around population GS in this study) from the rest of the HM—as a barrier to gene flow, as has been previously found in yew trees (Liu et al., 2013). Taken together, our analyses as well as previous work show geological features in the HM create significant barriers to gene flow and lead to discrete population genetic structure, but the specific patterns are likely to be idiosyncratic to different biomes and taxa.

In addition, by sequencing typical individuals of each species in the *G. hexaphylla* complex, our genomic data show that all samples of species co-occurring with *G. hexaphylla* cluster together based on their geographical origin rather than their morphological traits (taxonomic attribution). Thus, our results not only point to geographical structuring, but also highlight the need for taxonomic clarification in this species complex.

### Divergence correlates with uplift and climate change

The timing of geological events leading to the current topological conformation in the HM is still debated (Favre et al., 2015; Spicer et al., 2020), but most studies agree that at least some parts of the HM (in the east) have experienced local uplift in the HM during the Late Miocene to the Pliocene (Favre et al., 2015; Ding et al., 2020), and that this caused *in situ* diversification of many alpine groups (Xing and Ree, 2017; Muellner-Riehl et al., 2019; Ye et al., 2019). However, other studies suggest that the extent of Pleistocene climate fluctuation are a key factor causing divergence, rather than geological processes (Muellner-Riehl, 2019). The divergence between the two main lineages in the *G. hexaphylla* complex dated to the Pliocene, and correlated with the uplift of the Daxue Mountains, including Mt. Gonggar (7,556 m above sea level, a.s.l.), which may then have acted as barrier to dispersal. This is likely to be similar to *Pinus armandii* (Liu et al., 2019). Our ancestral area estimation also indicated that the *G. hexaphylla* complex originated in the central HM and then dispersed northward and southward, suggesting that the species occurred in the region of the Daxue Mountains and then experienced divergence associated with mountain uplift. However, climatic oscillations lead to variable connectivity among sky-islands in mountain systems, as previously shown in the HM (Deng et al., 2020) and considered in the Flickering Connectivity System’ proposed for the Andean flora (Flantua et al., 2019). Through vertical displacement as climate oscillates, some areas may be characterized by cycles of extinction and colonization, while other areas may be colonized anew. Thus, the dispersal from the central HM to other regions, as well as differentiation in isolation in each of these regions, may have been caused by climate oscillations. Therefore, the *G. hexaphylla* complex may bear the marks of a species-pump effect, as predicted by the *Mountain-Geobiodiversity Hypothesis* (Mosbrugger et al., 2018).

### Colonization from the HM to Mount Taibai

The *G. hexaphylla* complex is distributed across two biogeographically disjunct regions, namely the HM and the QM. The QM provides a natural boundary between northern and southern China, and served as a geographical and ecological barrier for species with low dispersal ability (Yan et al., 2010; Hu et al., 2021). Its isolation also promoted the divergence of some relict species (Shahzad et al., 2020). Mount Taibai, belonging to the QM, is the highest peak (3,500 m a.s.l.) in Central and East China and sits more than 400 km northeast of the HM. At its highest elevation it harbors an alpine flora including several *Gentiana* species (Ho and Pringle, 1995), for example the endemic *G. apiata* N.E. Brown (Ho and Liu, 2001). Our species distribution modelling showed that this region may have been suitable for *G. hexaphylla* since the LGM. As Mount Taibai was glaciated during the LGM (Rost, 1994; Zhang et al., 2016), *G. hexaphylla* individuals now occurring in this region are likely to be the result of uphill migration as glaciation receded. This colonization scenario would be consistent with climate change from cold and humid to warm and dry from 18 kya to present in the QM (Zhao et al., 2014). This inference is also supported by our pairwise *F*_ST_ values and the genetic clustering results, which both showed less genetic differentiation between population TB and the northern populations of the HM. This result indicates that *G. hexaphylla* is likely to have colonized the QM from the North HM. To our knowledge, our study is the first to show the dispersal directionality between the QM and the North HM, although at the genus level, *Gentiana* is known to match the *out-of-Tibet* hypothesis (Favre et al., 2016). Although more case studies are needed to evaluate the relative role of the different modes of assembly of the alpine biome in the QM, our results do improve our understanding of how *Gentiana*, and likely other alpine lineages, may have colonized these mountains.

## Conclusion

Using genomic data, this study recovered deep differentiation between populations of the *G. hexaphylla* complex along two sides of the Daxue Mountains in the Central HM, from where the complex originated. Divergence is likely to have been driven by a combination of mountain uplift, climatic fluctuations and geographical isolation. We also found that the QM were colonized from the HM by *G. hexaphylla* relatively recently, probably aided by changes in climatic conditions.

## Data availability statement

The data presented in the study are deposited in Dryad (doi: 10.5061/dryad.0gb5mkm08).

## Author contributions

P-CF collected the samples, analyzed the data, and wrote the manuscript. S-SS did lab work and prepared the tables and figures. PH and AF revised the manuscript. S-LC collected the samples. AT guided the analysis and revised the manuscript. All authors contributed to the article and approved the submitted version.

## Funding

This study was supported by the National Natural Science Foundation of China (grant no. 31600296) and Chinese Scholarship Council to P-CF. AF was supported by the German Science Foundation (Deutsche Forschungsgemeinschaft) project no. FA1117/1–2.

## Conflict of interest

The authors declare that the research was conducted in the absence of any commercial or financial relationships that could be construed as a potential conflict of interest.

## Publisher’s note

All claims expressed in this article are solely those of the authors and do not necessarily represent those of their affiliated organizations, or those of the publisher, the editors and the reviewers. Any product that may be evaluated in this article, or claim that may be made by its manufacturer, is not guaranteed or endorsed by the publisher.

## Footnotes

^1^ https://github.com/darencard/RADpipe

^2^ http://tree.bio.ed.ac.uk/software/tracer/

^3^ http://tree.bio.ed.ac.uk/software/figtree/

^4^ https://www.gbif.org
