# Supplementary material for: Population genomics reveal deep divergence and strong geographical structure in gentians in the Hengduan Mountains
Source: Front Plant Sci. 2022 Aug 25;13:936761. doi: 10.3389/fpls.2022.936761 (PMC9453878; doi:10.3389/fpls.2022.936761)
Supplement: Supplementary file 1 [file Data_Sheet_1.DOCX]

**Table S1** Information of ourgroups used for phylogenetic reconstruction.

| Species | Taxonomic treatment | GenBank no. |
| --- | --- | --- |
| *Gentiana tongolensis* | section *Microsperma* | MK251985 |
| *Gentiana trichotoma* | section *Frigida* | MN089577 |

**Table S2** Information of RAD-seq raw data in *Gentiana hexaphylla* complex.

| Sample | Raw Reads | Clean Reads | Effective Rate(%) | Q30(%) | GC Content(%) |
| --- | --- | --- | --- | --- | --- |
| fu60462 | 16,250,445 | 16,139,941 | 99.32 | 92.06 | 40.19 |
| fu60465 | 13,957,815 | 13,872,738 | 99.39 | 91.89 | 40.19 |
| fu60467 | 13,826,276 | 13,728,684 | 99.29 | 92.03 | 40.02 |
| fu60468 | 14,377,442 | 14,265,203 | 99.22 | 91.54 | 40.61 |
| fu604610 | 12,471,966 | 12,332,919 | 98.89 | 91.57 | 41.36 |
| fu604611 | 16,018,724 | 15,878,078 | 99.12 | 92.03 | 40.28 |
| fu60871 | 19,543,366 | 19,328,687 | 98.90 | 91.62 | 40.03 |
| fu60874 | 14,242,450 | 14,067,470 | 98.77 | 91.56 | 41.05 |
| fu60875 | 13,686,471 | 13,511,340 | 98.72 | 91.23 | 40.75 |
| fu60877 | 13,396,417 | 13,249,721 | 98.90 | 91.37 | 40.65 |
| fu60878 | 16,518,641 | 16,337,755 | 98.58 | 91.88 | 39.94 |
| fu60879 | 32,168,824 | 31,816,563 | 98.79 | 91.85 | 40.53 |
| fu61503 | 19,668,406 | 19,453,029 | 98.55 | 91.79 | 41.34 |
| fu61504 | 23,387,826 | 23,122,272 | 98.86 | 92.77 | 39.67 |
| fu61561 | 15,337,596 | 15,070,199 | 98.26 | 92.59 | 40.09 |
| fu61564 | 14,143,194 | 13,859,241 | 97.99 | 92.70 | 41.30 |
| fu61565 | 17,198,539 | 16,929,880 | 98.44 | 88.82 | 40.05 |
| fu61566 | 13,262,974 | 12,972,708 | 97.81 | 89.31 | 40.90 |
| fu71703 | 26,545,334 | 25,964,378 | 99.25 | 92.11 | 40.29 |
| fu71709 | 18,632,741 | 18,567,914 | 99.65 | 92.83 | 41.19 |
| fu717013 | 25,301,518 | 25,183,634 | 99.53 | 92.72 | 39.89 |
| fu717019 | 28,271,427 | 28,165,284 | 99.62 | 92.78 | 40.64 |
| fu717024 | 17,356,227 | 17,271,478 | 99.51 | 92.63 | 41.45 |
| fu717028 | 15,190,777 | 15,102,218 | 99.42 | 93.25 | 40.62 |
| fu71713 | 16,492,959 | 16,396,809 | 99.25 | 88.61 | 40.06 |
| fu71714 | 12,488,348 | 12,337,157 | 98.79 | 90.41 | 40.95 |
| fu717112 | 13,282,543 | 13,131,614 | 98.86 | 89.81 | 40.09 |
| fu717114 | 18,165,271 | 17,976,175 | 98.96 | 90.47 | 40.38 |
| fu717115 | 12,714,425 | 12,562,803 | 98.81 | 89.28 | 40.84 |
| fu717118 | 12,905,357 | 12,717,564 | 98.54 | 89.75 | 40.52 |
| fu717119 | 16,455,901 | 16,224,841 | 98.60 | 89.34 | 39.47 |
| fu71722 | 13,805,323 | 13,687,138 | 99.14 | 89.94 | 39.66 |
| fu71725 | 18,242,444 | 18,088,452 | 99.16 | 89.56 | 39.93 |
| fu717210 | 18,519,729 | 18,366,784 | 99.17 | 89.79 | 40.11 |
| fu717211 | 49,887,455 | 49,475,460 | 99.09 | 90.38 | 41.14 |
| fu717226 | 47,297,292 | 46,906,688 | 99.15 | 90.40 | 39.68 |
| fu717228 | 12,254,082 | 12,152,882 | 97.88 | 90.20 | 43.46 |
| fu71731 | 11,574,734 | 11,362,374 | 98.17 | 89.48 | 39.71 |
| fu71733 | 23,047,576 | 22,624,725 | 98.59 | 92.24 | 41.13 |
| fu71734 | 24,942,048 | 24,484,440 | 98.69 | 92.56 | 40.20 |
| fu71736 | 19,418,817 | 19,062,543 | 98.79 | 92.34 | 39.73 |
| fu71738 | 18,408,017 | 18,324,612 | 99.55 | 92.38 | 39.45 |
| fu717313 | 13,686,337 | 13,598,611 | 99.36 | 92.32 | 39.48 |
| fu71991 | 18,074,081 | 17,965,909 | 99.40 | 93.34 | 39.83 |
| fu71992 | 20,738,267 | 20,570,248 | 99.19 | 92.64 | 41.15 |
| fu71993 | 18,887,158 | 18,754,099 | 99.30 | 93.00 | 39.66 |
| fu71996 | 12,223,222 | 12,104,921 | 99.03 | 92.67 | 40.93 |
| fu71997 | 22,759,780 | 22,539,502 | 99.41 | 90.79 | 39.52 |
| fu71998 | 32,161,903 | 31,850,628 | 99.30 | 90.69 | 39.73 |
| fu72011 | 16,445,029 | 16,285,868 | 99.12 | 90.29 | 40.14 |
| fu72013 | 27,955,218 | 27,684,657 | 98.01 | 90.17 | 39.67 |
| fu72014 | 34,488,391 | 34,154,599 | 98.21 | 90.64 | 39.65 |
| fu72019 | 14,842,468 | 14,590,862 | 98.30 | 91.37 | 39.45 |
| fu720113 | 17,824,596 | 17,373,049 | 97.47 | 91.85 | 39.96 |
| fu720122 | 23,578,452 | 22,706,259 | 96.30 | 92.06 | 40.27 |
| fu72025 | 25,846,144 | 25,696,246 | 99.42 | 91.78 | 39.31 |
| fu72029 | 12,807,499 | 12,676,453 | 98.98 | 92.49 | 39.53 |
| fu720212 | 12,571,605 | 12,459,650 | 99.11 | 92.33 | 39.66 |
| fu720216 | 19,332,928 | 19,187,965 | 99.25 | 92.57 | 39.56 |
| fu720218 | 20,664,718 | 20,516,407 | 99.28 | 92.55 | 39.96 |
| fu720227 | 17,715,798 | 17,506,032 | 98.82 | 92.52 | 40.28 |
| fu72294 | 31,888,726 | 31,640,546 | 99.22 | 92.57 | 40.03 |
| fu72296 | 19,785,616 | 19,657,168 | 99.35 | 92.36 | 39.69 |
| fu72297 | 18,266,072 | 18,109,093 | 99.14 | 92.20 | 40.40 |
| fu72298 | 28,874,861 | 28,672,809 | 99.30 | 92.17 | 39.41 |
| fu72299 | 16,047,925 | 15,935,630 | 99.26 | 92.09 | 39.09 |
| fu722910 | 28,242,806 | 28,045,177 | 99.11 | 92.09 | 39.70 |
| fu80521 | 80,903,232 | 80,677,825 | 99.72 | 89.78 | 40.74 |
| fu80523 | 15,322,867 | 15,223,417 | 99.35 | 92.11 | 40.45 |
| fu80525 | 15,567,502 | 15,508,844 | 99.62 | 90.86 | 40.88 |
| fu80527 | 13,277,534 | 13,225,065 | 99.60 | 90.74 | 41.28 |
| fu80529 | 15,120,758 | 15,073,935 | 99.69 | 90.82 | 40.70 |
| fu805211 | 18,222,674 | 18,130,313 | 99.49 | 93.62 | 40.81 |
| fu80641 | 16,018,515 | 15,924,271 | 99.41 | 93.75 | 40.92 |
| fu80643 | 10,422,286 | 10,364,108 | 99.44 | 93.83 | 40.95 |
| fu80645 | 13,002,018 | 12,889,207 | 99.13 | 90.78 | 41.95 |
| fu80647 | 15,176,286 | 15,028,847 | 99.03 | 90.88 | 40.44 |
| fu80649 | 11,714,712 | 11,612,853 | 99.13 | 90.78 | 40.77 |
| fu806411 | 13,470,899 | 13,209,078 | 98.06 | 93.36 | 40.65 |
| fu806413 | 24,433,529 | 23,958,638 | 98.63 | 93.79 | 40.44 |
| fu806415 | 16,767,026 | 16,441,141 | 98.57 | 93.15 | 40.81 |
| fu80881 | 12,304,472 | 12,209,610 | 99.23 | 90.22 | 40.45 |
| fu80883 | 11,993,865 | 11,904,543 | 99.26 | 90.00 | 40.70 |
| fu80885 | 13,362,984 | 13,205,344 | 98.82 | 90.18 | 40.50 |
| fu80887 | 12,696,795 | 12,537,971 | 98.75 | 92.79 | 40.53 |
| fu80889 | 11,881,776 | 11,727,935 | 98.71 | 92.90 | 40.78 |
| fu808813 | 10,484,704 | 10,352,025 | 98.73 | 92.86 | 40.53 |
| fu808814 | 12,512,989 | 12,334,790 | 98.58 | 92.70 | 40.75 |
| fu808817 | 13,533,331 | 13,345,496 | 98.61 | 92.59 | 40.83 |
| fu900101 | 9,271,284 | 8,633,347 | 93.12 | 90.97 | 39.25 |
| fu900102 | 4,498,192 | 4,446,098 | 98.84 | 93.03 | 39.84 |
| fu900103 | 5,399,807 | 5,337,068 | 98.84 | 92.82 | 39.51 |
| fu900104 | 6,180,948 | 6,057,312 | 98 | 92.57 | 40.27 |
| fu900105 | 12,327,978 | 12,146,783 | 98.53 | 92.88 | 40.25 |
| fu900106 | 6,313,806 | 5,905,889 | 93.54 | 90.2 | 39.51 |

**Table S3** Weir and Cockerham’s *F*_ST_ between each pair of populations based on genomic SNPs in the *Gentiana hexaphylla* complex. The population codes with background of gray are from the South HM.

| P. | SD | LH | LHter | LHtet | LHvia | HYter | HYvia | HY | JZ | TB | KD | XC | DQ | GS | CY |
| --- | --- | --- | --- | --- | --- | --- | --- | --- | --- | --- | --- | --- | --- | --- | --- |
| SD | - |  |  |  |  |  |  |  |  |  |  |  |  |  |  |
| LH | -0.016 | - |  |  |  |  |  |  |  |  |  |  |  |  |  |
| LHter | 0.008 | -0.056 | - |  |  |  |  |  |  |  |  |  |  |  |  |
| LHtet | 0.266 | 0.152 | 0.115 | - |  |  |  |  |  |  |  |  |  |  |  |
| LHvia | 0.109 | 0.012 | 0.034 | 0.004 | - |  |  |  |  |  |  |  |  |  |  |
| HYter | 0.281 | 0.251 | 0.180 | 0.214 | 0.193 | - |  |  |  |  |  |  |  |  |  |
| HYvia | 0.244 | 0.174 | 0.126 | 0.154 | 0.077 | 0.035 | - |  |  |  |  |  |  |  |  |
| HY | 0.264 | 0.164 | 0.127 | 0.087 | 0.053 | 0.073 | -0.012 | - |  |  |  |  |  |  |  |
| JZ | 0.278 | 0.156 | 0.120 | 0.096 | 0.027 | 0.169 | 0.038 | 0.007 | - |  |  |  |  |  |  |
| TB | 0.308 | 0.195 | 0.176 | 0.120 | 0.148 | 0.320 | 0.196 | 0.114 | 0.086 | - |  |  |  |  |  |
| KD | 0.141 | 0.063 | 0.070 | 0.157 | 0.095 | 0.201 | 0.148 | 0.163 | 0.148 | 0.207 | - |  |  |  |  |
| XC | 0.314 | 0.241 | 0.284 | 0.300 | 0.421 | 0.373 | 0.321 | 0.367 | 0.359 | 0.521 | 0.149 | - |  |  |  |
| DQ | 0.288 | 0.268 | 0.259 | 0.454 | 0.368 | 0.409 | 0.415 | 0.443 | 0.464 | 0.531 | 0.152 | 0.015 | - |  |  |
| GS | 0.337 | 0.314 | 0.307 | 0.441 | 0.425 | 0.467 | 0.437 | 0.479 | 0.482 | 0.510 | 0.198 | 0.053 | 0.006 | - |  |
| CY | 0.313 | 0.312 | 0.274 | 0.438 | 0.384 | 0.425 | 0.429 | 0.445 | 0.481 | 0.541 | 0.193 | 0.011 | 0.074 | 0.118 | - |

**Table S4** Individual assignment based on genomic SNPs and plastid data in *Gentiana hexaphylla* complex. Individuals are named with population name and Arabic number. The “red’, “orange” and “pink” correspond to different clusters in Fig. 1, 2 & 3. The “HN1”, “HN2” and “HS” are three subclades in Fig. 1.

|  | Individual | Species | SNP | Plastid |
| --- | --- | --- | --- | --- |
| 1 | TB1 | *G. hexaphylla* | red | - |
| 2 | TB2 | *G. hexaphylla* | red | HN1 |
| 3 | TB3 | *G. hexaphylla* | red | HN1 |
| 4 | TB4 | *G. hexaphylla* | red | HN1 |
| 5 | TB5 | *G. hexaphylla* | red | HN1 |
| 6 | TB6 | *G. hexaphylla* | red | HN1 |
| 7 | HYter1 | *G. ternifolia* | red | HN2 |
| 8 | HYter2 | *G. ternifolia* | red | HN2 |
| 9 | HYter3 | *G. ternifolia* | red | HN2 |
| 10 | HYter6 | *G. ternifolia* | red | HN2 |
| 11 | HYter7 | *G. ternifolia* | red | HN2 |
| 12 | HYter8 | *G. ternifolia* | red | HN2 |
| 13 | HYvia1 | *G. viatrix* | red | - |
| 14 | HYvia13 | *G. viatrix* | red | - |
| 15 | HYvia22 | *G. viatrix* | red | - |
| 16 | HYvia3 | *G. viatrix* | red | HN2 |
| 17 | HYvia4 | *G. viatrix* | red | - |
| 18 | HYvia9 | *G. viatrix* | red | HN2 |
| 19 | HY12 | *G. hexaphylla* | red | HN1 |
| 20 | HY16 | *G. hexaphylla* | red | HN2 |
| 21 | HY18 | *G. hexaphylla* | red | - |
| 22 | HY27 | *G. hexaphylla* | red | HN1 |
| 23 | HY5 | *G. hexaphylla* | red | - |
| 24 | HY9 | *G. hexaphylla* | red | HN2 |
| 25 | JZ10 | *G. hexaphylla* | red | HN1 |
| 26 | JZ4 | *G. hexaphylla* | red | HN2 |
| 27 | JZ6 | *G. hexaphylla* | red | - |
| 28 | JZ7 | *G. hexaphylla* | red | - |
| 29 | JZ8 | *G. hexaphylla* | red | - |
| 30 | JZ9 | *G. hexaphylla* | red | HN1 |
| 31 | SD2 | *G. hexaphylla* | red | HN1 |
| 32 | SD8 | *G. hexaphylla* | red | HN1 |
| 33 | SD10 | *G. hexaphylla* | orange | - |
| 34 | SD11 | *G. hexaphylla* | orange | HS |
| 35 | SD5 | *G. hexaphylla* | orange | HS |
| 36 | SD7 | *G. hexaphylla* | orange | - |
| 37 | LH1 | *G. hexaphylla* | orange | HS |
| 38 | LH3 | *G. hexaphylla* | orange | HS |
| 39 | LH8 | *G. hexaphylla* | orange | HS |
| 40 | LH13 | *G. hexaphylla* | red | HN1 |
| 41 | LH4 | *G. hexaphylla* | red | - |
| 42 | LH6 | *G. hexaphylla* | red | HN1 |
| 43 | LHter24 | *G. ternifolia* | orange | - |
| 44 | LHter28 | *G. ternifolia* | orange | HS |
| 45 | LHter9 | *G. ternifolia* | orange | HS |
| 46 | LHter13 | *G. ternifolia* | red | HN1 |
| 47 | LHter19 | *G. ternifolia* | red | - |
| 48 | LHter3 | *G. ternifolia* | red | HN1 |
| 49 | LHvia11 | *G. viatrix* | orange | - |
| 50 | LHvia10 | *G. viatrix* | red | - |
| 51 | LHvia2 | *G. viatrix* | red | - |
| 52 | LHvia26 | *G. viatrix* | red | HN1 |
| 53 | LHvia28 | *G. viatrix* | red | HN1 |
| 54 | LHvia5 | *G. viatrix* | red | HN1 |
| 55 | LHtet12 | *G. tetraphylla* | red | HN1 |
| 56 | LHtet14 | *G. tetraphylla* | red | - |
| 57 | LHtet15 | *G. tetraphylla* | red | - |
| 58 | LHtet18 | *G. tetraphylla* | red | - |
| 59 | LHtet19 | *G. tetraphylla* | red | HN1 |
| 60 | LHtet3 | *G. tetraphylla* | red | HN1 |
| 61 | LHtet4 | *G. tetraphylla* | red | - |
| 62 | KD1 | *G. hexaphylla* | red | HN1 |
| 63 | KD5 | *G. hexaphylla* | red | HN1 |
| 64 | KD4 | *G. hexaphylla* | pink+red | KD |
| 65 | KD7 | *G. hexaphylla* | pink+red | - |
| 66 | KD8 | *G. hexaphylla* | pink+red | - |
| 67 | KD9 | *G. hexaphylla* | pink+red | HS |
| 68 | XC3 | *G. hexaphylla* | pink | - |
| 69 | XC4 | *G. hexaphylla* | pink | - |
| 70 | XC1 | *G. hexaphylla* | pink | HS |
| 71 | XC4 | *G. hexaphylla* | pink | - |
| 72 | XC5 | *G. hexaphylla* | pink | HS |
| 73 | XC6 | *G. hexaphylla* | pink | - |
| 74 | DQ1 | *G. hexaphylla* | pink | HS |
| 75 | DQ11 | *G. hexaphylla* | pink | HS |
| 76 | DQ3 | *G. hexaphylla* | pink | HS |
| 77 | DQ5 | *G. hexaphylla* | pink | - |
| 78 | DQ7 | *G. hexaphylla* | pink | - |
| 79 | DQ9 | *G. hexaphylla* | pink | HS |
| 80 | GS1 | *G. hexaphylla* | pink | HS |
| 81 | GS11 | *G. hexaphylla* | pink | - |
| 82 | GS13 | *G. hexaphylla* | pink | HS |
| 83 | GS15 | *G. hexaphylla* | pink | HS |
| 84 | GS3 | *G. hexaphylla* | pink | HS |
| 85 | GS5 | *G. hexaphylla* | pink | HS |
| 86 | GS7 | *G. hexaphylla* | pink | HS |
| 87 | GS9 | *G. hexaphylla* | pink | HS |
| 88 | CY1 | *G. hexaphylla* | pink | HS |
| 89 | CY13 | *G. hexaphylla* | pink | HS |
| 90 | CY14 | *G. hexaphylla* | pink | - |
| 91 | CY17 | *G. hexaphylla* | pink | HS |
| 92 | CY3 | *G. hexaphylla* | pink | - |
| 93 | CY5 | *G. hexaphylla* | pink | HS |
| 94 | CY7 | *G. hexaphylla* | pink | HS |
| 95 | CY9 | *G. hexaphylla* | pink | HS |


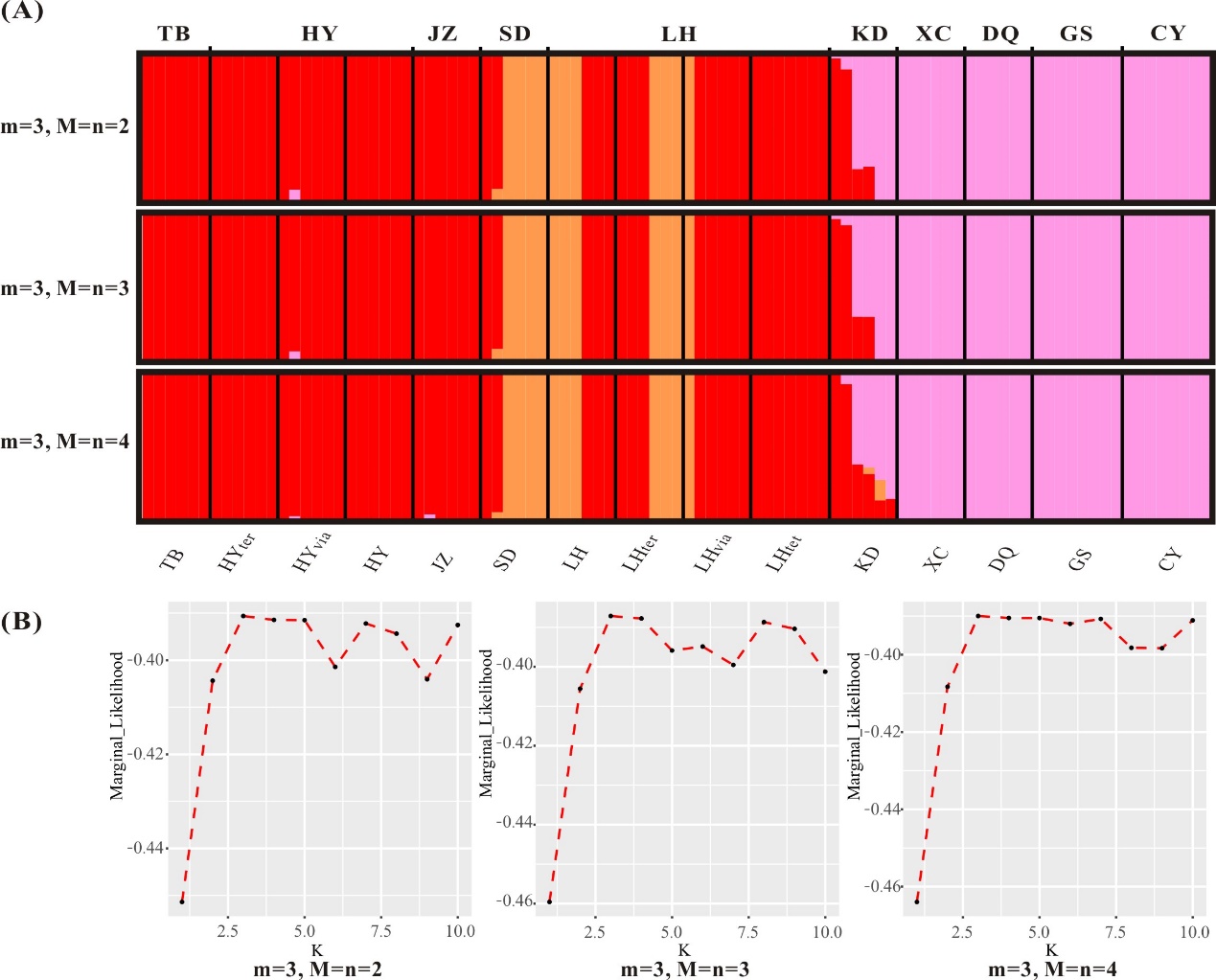


**Fig. S1** Results in FastStructure based on three sets of data in *Gentiana hexaphylla* complex. (A) Bar plots showing probabilities of ancestral clusters of each sample at K = 3. The name of each population and sampling site is shown below and above the bar plot, respectively. (B) The marginal likelihood values verses number of clusters (K).


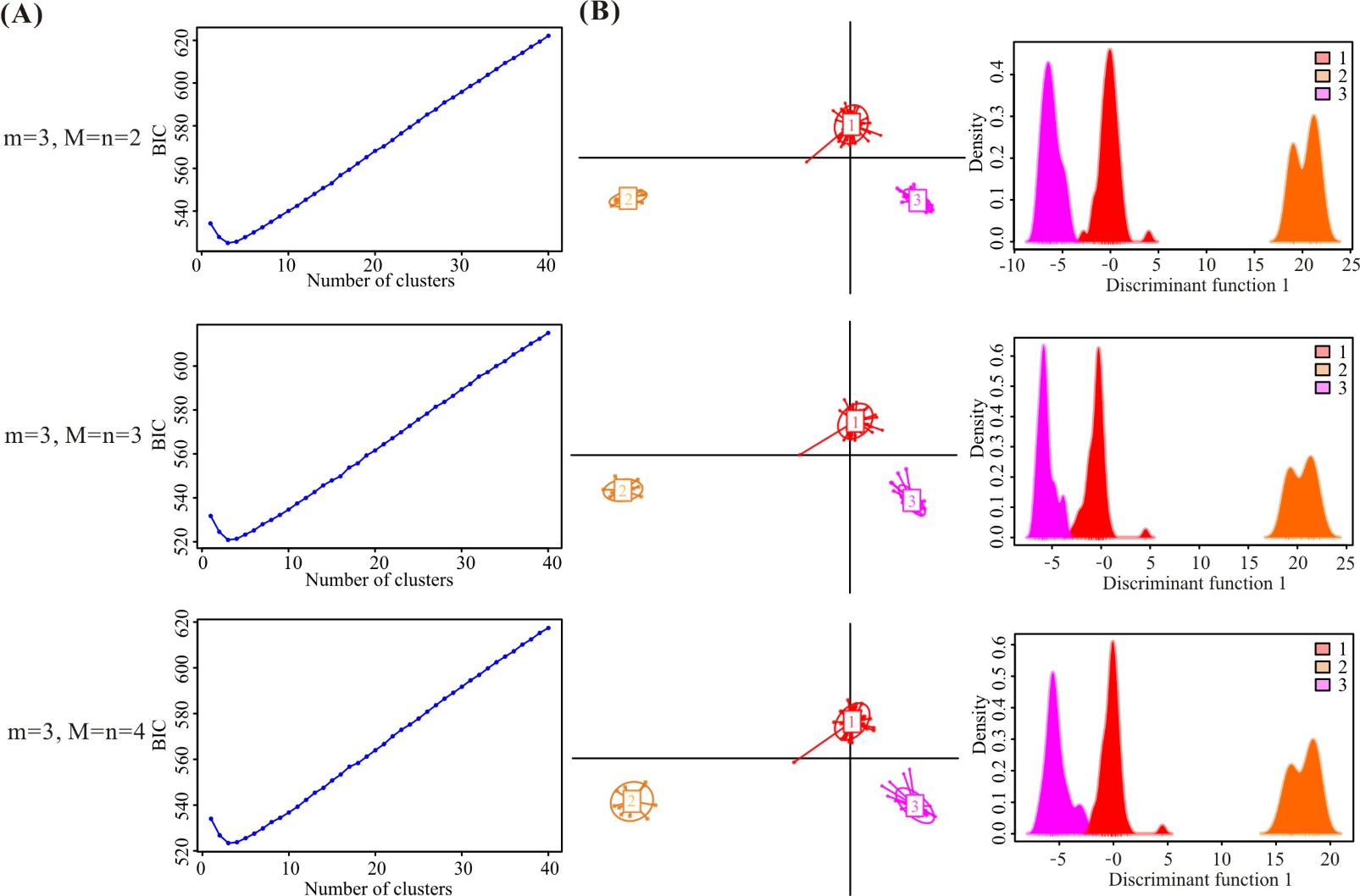


**Fig. S2** Results from DAPC based on three sets of data in *Gentiana hexaphylla* complex. (A) The value of BIC verses number of clusters (K). (B) Scatterplot of DAPC analysis. Each dot represents an individual. Insets show histograms of discriminant analysis eigenvalues.


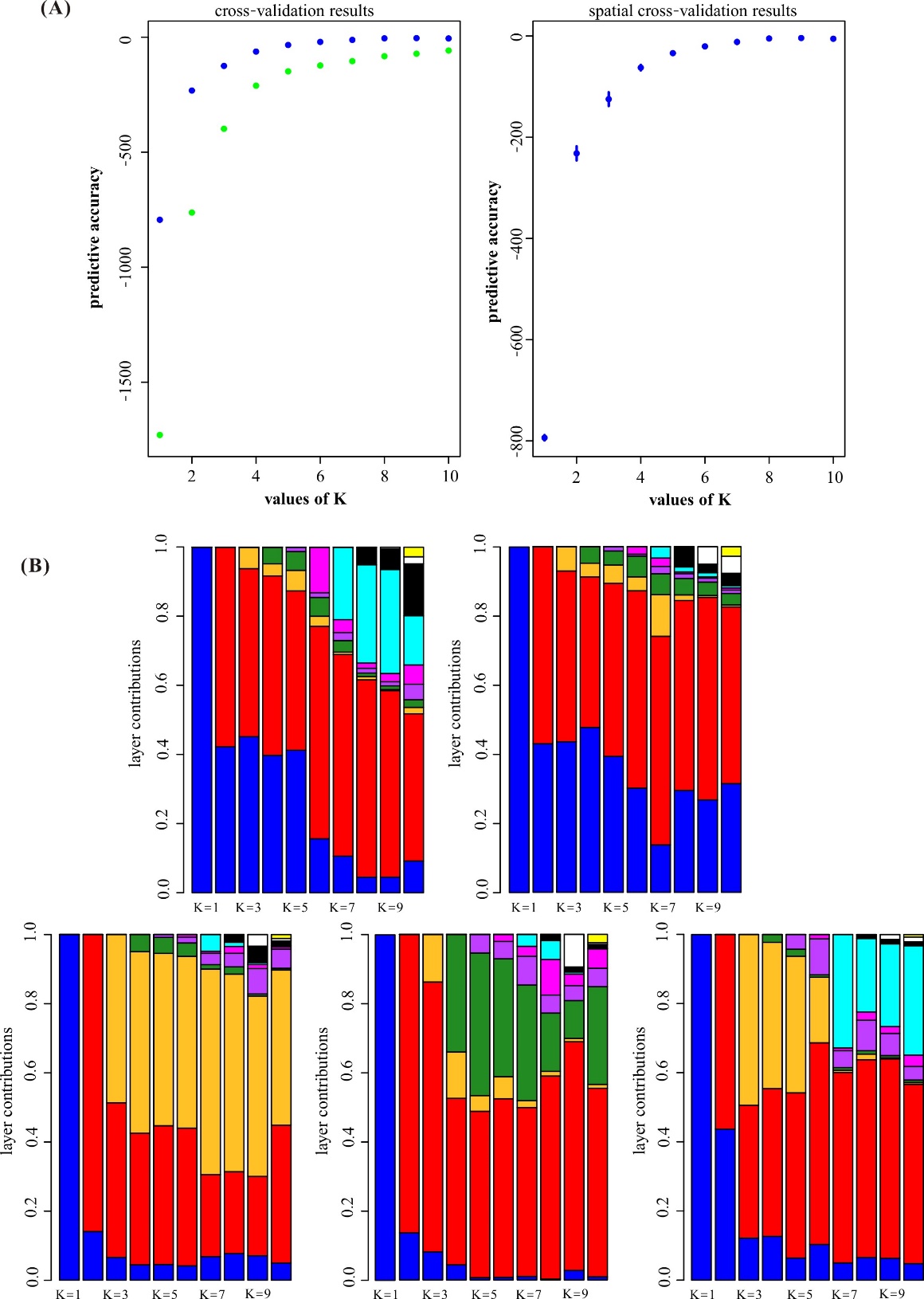


**Fig. S3** The results of *Gentiana hexaphylla* complex conStruct models with K from 1 to 10. (A) The cross-validation result. The blue points represent the predictive accuracy for the spatial model, and the green for the non-spatial model. (B) Covariance contribution of each layer (K) in the five replicates.

**
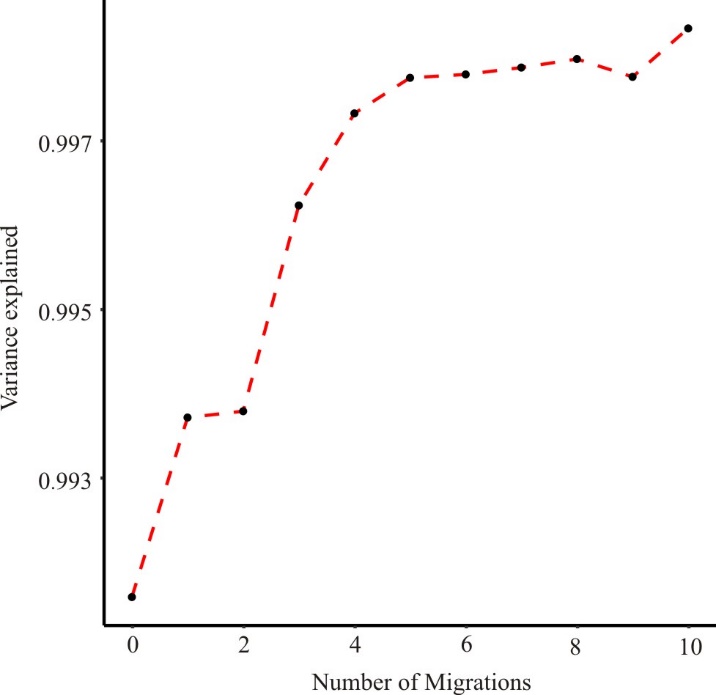
**

**Fig. S4** The variance explained by differing migration numbers simulated in Treemix.


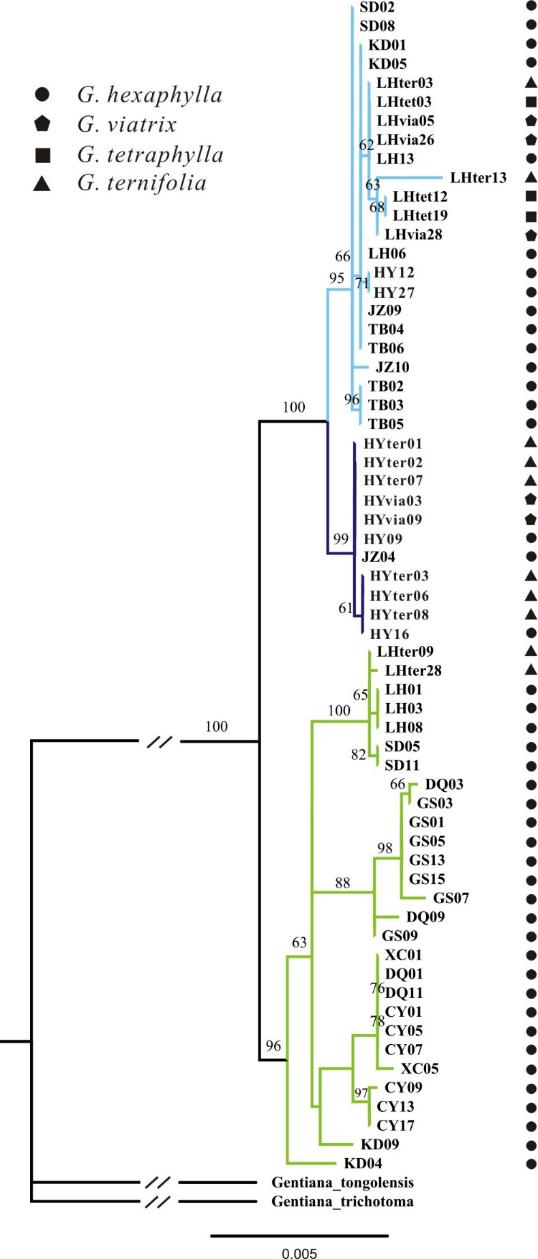


**Fig. S5** ML tree based on plastid datasets in the *Gentiana hexaphylla* complex. Shapes indicate different species: circle, *G. hexaphylla*; pentagon, *G. viatrix*; square, *G. teterphylla*; triangle, *G. ternifolia*. Phylogenetic support values for maximum likelihood are shown at nodes only when they above 60% bootstrap support. The double slash (//) symbolizes an artificial shortening of this branch. Dark shapes in panel B and C indicate different species: circle, *G. hexaphylla*; pentagon, *G. viatrix*; square, *G. teterphylla*; triangle, *G. ternifolia*.


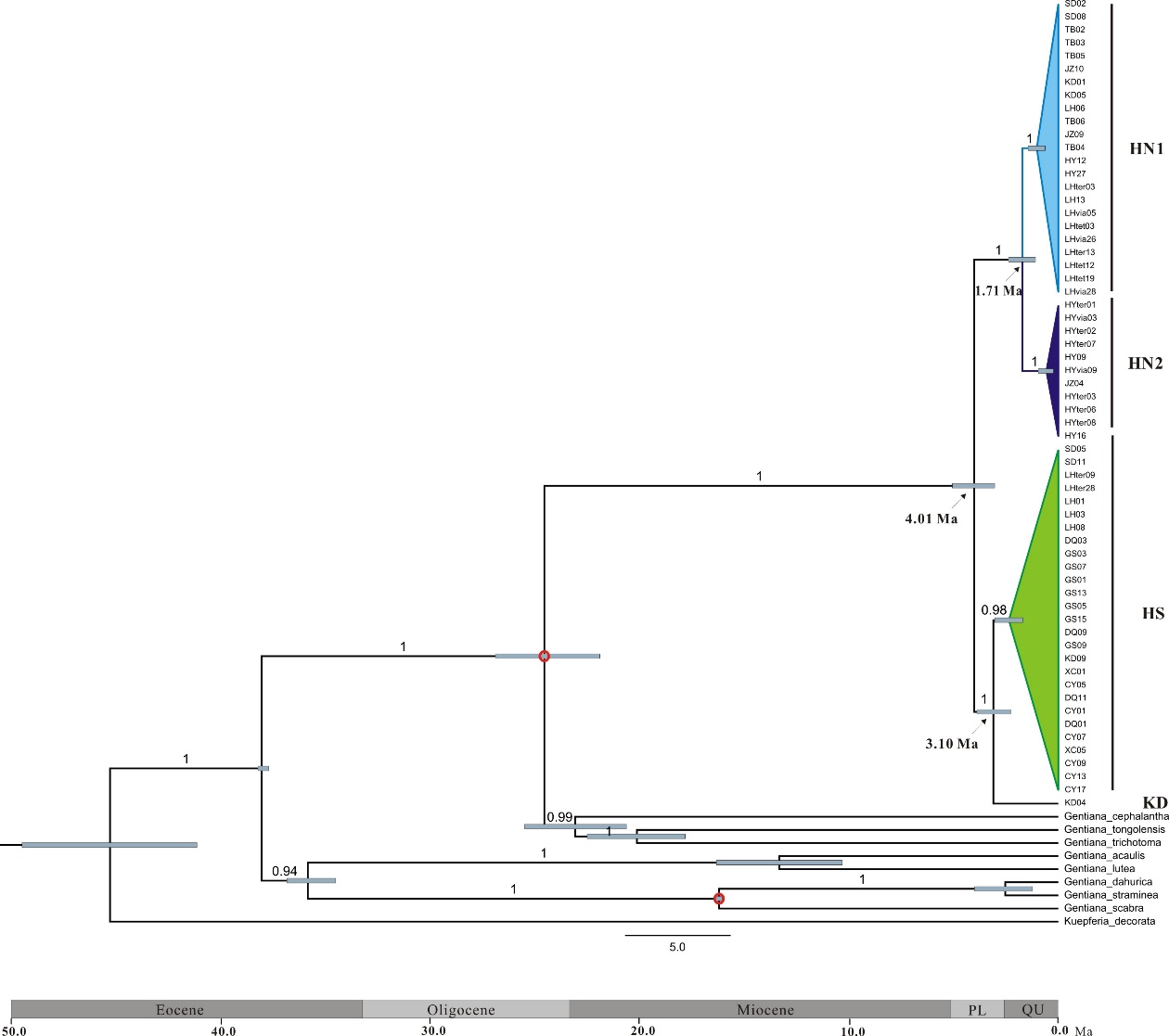


**Fig. S6** Majority rule consensus phylogenetic tree of plastid datasets in the *Gentiana hexaphylla* complex based on Bayesian inference using plastid sequences. Numbers on the branches indicate Bayesian posterior probabilities. Node age estimates are marked with black arrows. Grey bars represent 95% highest posterior densities. Two constrained nodes are showed using red circles.
